# Pre- and post-operative anti-PD-L1 plus anti-angiogenic therapies in mouse breast or renal cancer models of micro- or macro-metastatic disease

**DOI:** 10.1038/s41416-018-0297-1

**Published:** 2018-11-30

**Authors:** Florence T. H. Wu, Ping Xu, Annabelle Chow, Shan Man, Janna Krüger, Kabir A. Khan, Marta Paez-Ribes, Elizabeth Pham, Robert S. Kerbel

**Affiliations:** 1 Department of Medical Biophysics, University of Toronto, Toronto, ON Canada; 2 Biological Sciences Platform, Sunnybrook Research Institute, Toronto, ON Canada; 3 Present Address: Department of Oncology, University of Cambridge, Hutchison/MRC Research Centre, Cambridge, UK; 4 Present Address: Amgen Discovery Research, South San Francisco, CA USA

**Keywords:** Targeted therapies, Tumour angiogenesis, Tumour immunology, Metastasis, Cancer immunotherapy

## Abstract

**Background:**

There are phase 3 clinical trials underway evaluating anti-PD-L1 antibodies as adjuvant (postoperative) monotherapies for resectable renal cell carcinoma (RCC) and triple-negative breast cancer (TNBC); in combination with antiangiogenic VEGF/VEGFR2 inhibitors (e.g., bevacizumab and sunitinib) for metastatic RCC; and in combination with chemotherapeutics as neoadjuvant (preoperative) therapies for resectable TNBC.

**Methods:**

This study investigated these and similar clinically relevant drug combinations in highly translational preclinical models of micro- and macro-metastatic disease that spontaneously develop after surgical resection of primary kidney or breast tumours derived from orthotopic implantation of murine cancer cell lines (RENCA^luc^ or EMT-6/CDDP, respectively).

**Results:**

In the RENCA^luc^ model, adjuvant sunitinib plus anti-PD-L1 improved overall survival compared to either drug alone, while the same combination was ineffective as early therapy for unresected primary tumours or late-stage therapy for advanced metastatic disease. In the EMT-6/CDDP model, anti-PD-L1 was highly effective as an adjuvant monotherapy, while its combination with paclitaxel chemotherapy (with or without anti-VEGF) was most effective as a neoadjuvant therapy.

**Conclusions:**

Our preclinical data suggest that anti-PD-L1 plus sunitinib may warrant further investigation as an adjuvant therapy for RCC, while anti-PD-L1 may be improved by combining with chemotherapy in the neoadjuvant but not the adjuvant setting of treating breast cancer.

## Background

The number of the Food and Drug Administration (FDA)-approved indications for immune checkpoint inhibitors that target the programmed death-1/programmed death-ligand 1 (PD-1/PD-L1) pathway (i.e., pembrolizumab, nivolumab, atezolizumab, avelumab, and durvalumab) in clinical oncology has rapidly expanded in the last 3 years and is now approaching a dozen distinct solid tumour types to include skin, lung, kidney, bladder, head and neck, lymphocytic, liver, and gastric cancers (Suppl Table 1). Yet, based on phase 3 clinical trial data, the clinical response rates for PD-1/PD-L1 pathway inhibitors when used as monotherapies have only been in the range of 20-44%^1^—thus, the need for combination therapies remains.

In 2015, the FDA granted regular approval to the use of nivolumab as treatment for advanced metastatic RCC (mRCC) after progression on prior antiangiogenic therapies (including sunitinib, axitinib, bevacizumab, etc.) based on a successful randomised phase 3 clinical trial (“CheckMate-025^2^). Then in 2017, the FDA granted accelerated approval to the use of nivolumab in previously sorafenib-treated hepatocellular carcinoma based on a promising phase 1/2 clinical trial (“CheckMate-040”^3^). Sunitinib, axitinib, and sorafenib are small-molecule tyrosine kinase inhibitors (TKIs) that inhibit the endothelial vascular endothelial growth factor receptor-2 (VEGFR2) among its multitude of targets, while bevacizumab is an antibody against the VEGF-A (often simplified as “VEGF”) ligand. Beyond these approved sequential anti-PD-1-after-antiangiogenic combinations, there are at least five ongoing randomised phase 3 trials evaluating concurrent anti-PD-L1-plus-antiangiogenic combinations in late-stage (inoperable) RCC, non-small-cell lung cancer, and ovarian cancer (Suppl. Table 2). Of the 11 ongoing randomised phase 3 trials evaluating anti-PD-L1 agents in earlier-staged cancers as adjuvant (postoperative) or neoadjuvant (preoperative) therapies, there is also one ovarian cancer trial that includes an antiangiogenic drug in the investigational drug combination (Suppl. Table 3).

The biological rationale supporting various combinations of immunotherapeutics and antiangiogenic therapies have previously been reviewed.^4–9^ Aside from its angiogenic functions, VEGF also acts as an immunosuppressive factor within the tumour microenvironment by inhibiting dendritic cell (DC) maturation,^10^ inducing DC expression of PD-L1,^11^ downregulating endothelial cell (EC) adhesion molecules that facilitate the extravasation of cytotoxic T lymphocytes (CTLs) into tumours,^4^ and promoting infiltration of tumours by T regulatory cells (Tregs).^12^ Thus, antiangiogenic VEGF/VEGFR2 pathway inhibitors may act as de facto immunotherapeutics by relieving these immunosuppressive effects to enhance CTL infiltration and/or activity within tumours.^13,14^

Most of aforementioned clinical trials investigating anti-PD-L1 plus antiangiogenic drug combinations in human patients were designed and initiated without extensive prior modelling in preclinical animal studies. To our knowledge, only four preclinical studies have investigated the co-targeting of VEGF-A and PD-1,^15^ co-targeting of VEGFR2 plus PD-1,^16^ co-targeting of VEGFR2 plus PD-L1,^17^ as well as triple targeting of VEGF, Angiopoietin-2, and PD-1^18^—and these have relied heavily on primary tumour models (of melanoma,^18^ pancreatic neuroendocrine tumours (PNETs),^17^ and colorectal^15,16,18^ and breast cancer^17,18^) and only utilised one metastatic model (of breast cancer^18^). Among these, the melanoma and colorectal models all involved subcutaneous (heterotopic) implantations; only PNET and breast cancer models were transgenic/orthotopic models. Moreover, none of these preclinical models involved surgical resection of primary tumours to explicitly evaluate these therapies in pre- or post-operative settings—an important gap that we sought to fill with our current study.

For antiangiogenic drugs, our lab has repeatedly shown that simple primary tumour models tend to over-predict clinical efficacy compared to more sophisticated metastatic tumour models in which orthotopically grown primary tumours are surgically resected so that the VEGF/VEGFR2 pathway inhibitors can be administered at clinically relevant time points to either treat microscopic metastatic disease in the perioperative setting^19–21^ or macroscopic metastases in the advanced disease setting.^22^ Here are two examples. The first example relates to how sunitinib alone or in combination with chemotherapeutics as an experimental therapy for advanced metastatic breast cancer has failed to meet its primary endpoint in four major phase III clinical trials.^23^ These clinical trials were preceded by promising preclinical data showing sunitinib-induced growth inhibition of primary tumours in three breast cancer models (including a transgenic, a chemically induced, and a xenograft model), as well as delayed progression of artificial bone metastases (modelled by bone colonisation after left ventricular inoculation of tumour cells).^24^ Retrospectively, our lab showed that the lack of survival benefits in clinical trials can in fact be accurately recapitulated preclinically in mice if sunitinib is administered after the establishment of spontaneous macroscopic metastatic disease (in the lungs and lymphatics, etc.) following surgical resection of primary breast tumours.^22^ Moreover, our recent work revealed one potential explanation for this discrepancy in response to antiangiogenic therapy between metastases versus their originating primary tumours—lung metastases often co-opt pre-existing alveolar capillaries for vascularisation rather than rely on de novo sprouting angiogenesis, unlike primary orthotopic tumours.^25^ Another example relates to the minimal benefits or outright failures of VEGF/VEGFR2 pathway inhibitors in multiple phase III clinical trials as adjuvant therapies to treat minimally residual disease or microscopic metastases postoperatively, even though the very same agents have shown clinical activity and have received regulatory approval for clinical use in the advanced metastatic disease settings of the same cancer types (e.g., bevacizumab for colorectal cancer,^26–29^ sunitinib for RCC^30–33^). Our lab—by performing surgical resection of primary orthotopic tumours (including breast, renal, and colorectal tumours) and administering antiangiogenic drugs in the immediately postoperative (or preoperative) setting to model clinical adjuvant (or neoadjuvant) therapies—had prospectively predicted the lacklustre performance of VEGF/VEGFR2 inhibitors in such early-stage disease settings.^19,20^ Using these same models, our more recent work has suggested potential strategies for improving the effectiveness of VEGF/VEGFR2 inhibitors as perioperative therapies through combination with Angiopoietin/Tie2 pathway-targeted agents.^34,35^

Hence, the objective of this study was to extend the use of our highly translational models of postsurgical metastatic disease to the preclinical evaluation of anti-PD-L1 plus antiangiogenic drug combinations in the context of renal and breast cancer.

Firstly, using a kidney cancer model that involves orthotopic implantation of the murine cancer cell line, RENCA^luc^, followed later by nephrectomies, we evaluated the combination of sunitinib+anti-PD-L1 vs. anti-VEGF+anti-PD-L1 in the adjuvant therapy setting, and further compared the sunitinib+anti-PD-L1 combination as primary tumour treatment vs. adjuvant treatment vs. late-stage treatment of advanced metastatic disease. These combinations were chosen because both sunitinib and bevacizumab (given with interferon-alpha) are FDA-approved first-line therapies for metastatic RCC.^36^ Recently, the FDA made the somewhat controversial decision to further approve sunitinib as an adjuvant therapy for patients with locoregional RCC at high risk of recurrence post nephrectomy.^37–39^

Secondly, using a breast cancer model that involves orthotopic implantation of the murine cancer cell line, EMT-6/CDDP, followed later by mastectomies, we evaluated anti-VEGF+anti-PD-L1 vs. paclitaxel (chemotherapy)+anti-PD-L1 vs. anti-VEGF+paclitaxel+anti-PD-L1 in the adjuvant as well as neoadjuvant therapy settings. Bevacizumab given with chemotherapy remains an approved treatment in Europe and Asia, albeit not in North America, for patients with metastatic breast cancer.^36^ No antiangiogenic drugs have yet been approved as adjuvant or neoadjuvant treatment for early-stage breast cancer.

## Materials and methods

### Cell culture

The parental murine breast cancer cell line, EMT-6/P, as well as its cisplatin-resistant variant, EMT-6/CDDP, which was originally derived in the laboratory of Dr. Beverly Teicher,^40^ were provided to the laboratory of Dr. Robert Kerbel in 1991. RENCA^luc^ is a variant of the VHL-wild-type murine kidney cancer cell line, RENCA, that was transfected with the luciferase-tagged gene, as described here,^41^ in Dr. Kerbel’s laboratory in 2009. EMT-6/CDDP and RENCA^luc^ were passaged in Dulbecco's modified Eagle's medium (DMEM) high-glucose media with 5% foetal bovine serum (FBS) and RPMI-1640 media with 5% FBS, respectively, under 37 ^o^C, 21% O_2_, and 5% CO_2_. All cell lines were tested quarterly for mycoplasma (MycoAlert kit; Lonza) and last screened for viruses by PCR testing in 2016 (Mouse Essential CLEAR panel, Charles River Research).

### Syngeneic tumour studies

To set up the kidney cancer models, 10^5^ RENCA^luc^ cells suspended in 3 μL of serum-free media (SFM) were implanted orthotopically into the left renal subcapsule of 6-week-old male or female BALB/cJ mice. Resection of primary renal tumours, by way of complete nephrectomy of the left kidney, occurred on day 7 post implantation. Adjuvant therapy experiments involved starting sunitinib and anti-VEGF therapies at 10 days post implantation (DPI) and anti-PD-L1 therapy at 11 DPI. The late-stage therapy experiment—treating postsurgical advanced metastatic disease—involved starting sunitinib and anti-PD-L1 treatments at 14 and 15 DPI, respectively. In the one experiment where primary kidney tumours were not resected, sunitinib and anti-PD-L1 treatments began at 5 and 6 DPI, respectively. Serial whole-body bioluminescent imaging was performed using either an IVIS 200 system (PerkinElmer; 60 s, f/1, bin: 8, field of view (FOV) 23.2 cm) or IVIS Lumina II system (60 s, f/1, bin: 4, FOV 12.5 cm). Total fluxes (photons/s) were quantified using Living Image software (v2.50.1, Xenogen). Survival endpoints were primarily laboured breathing, abdominal distension, or ≥20% weight loss.

To set up the breast cancer models, 2 × 10^5^ EMT-6/CDDP cells suspended in 50 μL of SFM were orthotopically implanted into the right inguinal mammary fat pad of 6-week-old female BALB/cJ mice (Jackson Labs). In the adjuvant therapy experiments, primary breast tumours averaging around 500 mm^3^ were resected at 11 or 12 DPI and adjuvant treatments began 1 day later. In the neoadjuvant therapy experiment, mice were treated from days 6 to 10 post implantation, before primary breast tumours were resected on day 11. The one group in the neoadjuvant experiment that also received adjuvant therapy was restarted on anti-VEGF therapy on day 13. Survival endpoints included laboured breathing, ascites, ulceration of post-surgically regrown primary tumours, hindlimb paralysis, and ≥20% weight loss.

Randomisation of mice into treatment groups occurred immediately before initiation of systemic neoadjuvant or adjuvant or late-stage therapies. Kaplan–Meier statistical analyses of overall survival were performed on GraphPad Prism software (San Diego, USA).

### In vivo treatments

Paclitaxel (Accord Healthcare Inc., DIN: 02391465) was administered at 30 mg/kg, every 2 weeks, intraperitoneally (IP); phosphate-buffered saline (PBS) served as its control. Sunitinib malate (LC Laboratories; administered at 60 mg/kg daily) and its vehicle, formulated as previously described,^42^ were administered by oral gavage (PO). The therapeutic anti-VEGF agent—a mouse IgG2a antibody against mouse/human VEGF (Genentech, clone B20-4.1.1)—was administered at 5 mg/kg, 2×/week, IP. The two therapeutic antibodies to mouse PD-L1 (also known as CD274 or B7-H1) were a mouse IgG1 raised in PD-L1 knockout mice with a C57BL/6 genetic background (Genentech, clone 6E11; administered at 5 mg/kg, 2×/week, IP) and a rat IgG2b monoclonal antibody (BXCell InVivoMAb #BE0101, clone 10F.9G2; administered at 100 μg/dose, 2×/week, IP)—both antibodies block PD-L1:PD-1 and PD-L1:B7.1 (CD80) interactions. The corresponding InVivoMAb controls from BioXCell were: mouse IgG2a isotype control (#BE0085, clone C1.18.4, unknown specificity), mouse IgG1 isotype control (#BE0083, clone MOPC-21, unknown specificity), and rat IgG2b isotype control (#BE0090, clone LTF-2, anti-keyhole limpet haemocyanin).

### Ex vivo tumour dissociation and flow cytometry

Dissected tumours were minced into 2–4 mm pieces and incubated in enzyme digestion buffer (including collagenase II and IV and DNase I) for 40 min at 37 °C. After stopping digestion with 5% FBS/PBS on ice, tumour cell suspensions were filtered through 70 μm pore nylon cell strainers and centrifuged. Cell pellets were then resuspended and incubated in RBC lysis buffer (5 min, room temperature (RT)), washed twice in PBS, and resuspended in cold 2% FBS/PBS. Samples of 10^6^ cells/100 μL were incubated with premixed cocktails of staining antibodies—PE rat anti-mouse PD-L1 (clone 10F.9G2, BioLegend #124308), PerCP rat anti-mouse CD45 (clone 30-F11, BD #557235), PE-Cy7 rat anti-mouse Flk1 (clone AVAS12α1, BD #561259), APC rat anti-mouse CD31 (clone MEC13.3, BD #551262), APC-Cy7 rat anti-mouse CD45 (clone 30-F11, BD #557659), Alexa Fluor 700 hamster anti-mouse CD3e (clone 500A2, BD #557984), PE-Dazzle 594 rat anti-mouse CD4 (clone GK1.5, BioLegend #100456), and PE-Cy7 rat anti-mouse CD8a (clone 53-6.7, BioLegend #100722)—for 30 min on ice, in the dark, on a shaker. Stained samples were then washed in 2 mL of FACS buffer and resuspended to 100 μL. A viability stain, 4′,6-diamidino-2-phenylindole (DAPI), was applied to samples 15 min before flow cytometry (≥10^4^ cells).

### In vitro tumour cell expression of PD-L1

Approximately 80% confluent tumour cells in 6-well plates were switched to DMEM+2% FBS before undergoing a 16-h in vitro stimulation by recombinant mouse interferon-γ (R&D #485-MI). Stimulated cells were then washed in PBS, trypsinised, collected with DMEM+5% FBS, centrifuged at 4 ^o^C, and washed in 3 mL of FACS buffer twice (PBS+2% FBS), before suspending in FACS buffer. Samples of 10^6^ cells/100 μL were incubated with staining antibodies—PE-conjugated rat IgG2b, κ-anti-mouse PD-L1 (clone 10F.9G2, BioLegend #124308) or PE-conjugated rat IgG2b, κ-isotype control (eBioscience #12-4032-82)—at 1:150 for 30 min on ice, in the dark, on a shaker. Stained samples were then washed in 2 mL of FACS buffer and resuspended to ~100 μL. The 7-aminoactinomycin D viability stain was applied to samples 5 min before flow cytometry (≥10^4^ cells).

### Immunohistochemistry (IHC) staining of mouse PD-L1

The 5 μm-thick frozen mouse sections were air-dried for 20 min at RT, acetone fixed (5 min, RT), and air-dried. After peroxidase quenching with 1% aqueous H_2_O_2_ in methanol for 15 min and a rinse in tris-buffered saline with Tween (TBST), sections were blocked in protein block solution (Dako #X0909) with 10% donkey serum (30 min, RT), then incubated with the primary rabbit antibody to mouse PD-L1 (Genentech #GEN130-47-4; diluted to 10 μg/mL in antibody diluent (Dako #S3022)) in a humidity chamber at 4 °C overnight. Then, a biotinylated donkey anti-rabbit IgG secondary antibody (Jackson Immunoresearch #711-065-152; 1:200 in antibody diluent) was applied at RT for 30 min. Detection involved an ABC-HRP kit (VECTASTAIN Elite # PK-6100), a metal enhanced DAB substrate kit (Thermo Scientific #34065), and haematoxylin counterstaining. Bright-field images were taken with a Leica DM LB2 microscope and DFC 300 FX camera.

### Immunofluorescence (IF) staining of mouse PD-L1 and mouse endoglin

The 5 μm-thick frozen mouse sections were air-dried for 20 min at RT, acetone fixed (5 min, RT), and air-dried. Sections were blocked in protein block solution (Dako #X0909) with 5% of donkey and goat serum (30 min, RT), then incubated at 4 °C overnight with two primary antibodies, a rabbit anti-mouse PD-L1 (Genentech #GEN130-47-4; 10 μg/mL) and a rat anti-mouse endoglin/CD105 (clone 6J9, Abcam #ab56298; 0.5 μg/mL). Rabbit mAb IgG XP® isotype (Cell Signalling Technology #3900; 10 μg/mL) served as negative control for GEN130-47-4. Secondary antibodies, FITC goat anti-rabbit IgG (Vector Laboratories #FI-1000; 1:120) and Cy3 donkey anti-rat IgG (Cedarlane #712-165-153; 1:120), were then applied, followed by DAPI (Invitrogen #D3571; 0.5 mg/mL; 10 min, RT). Fluorescent images were taken with a Zeiss Axioplan 2 fluorescent microscope with AxioCam MRc camera.

## Results

### Co-administrating anti-PD-L1 improves adjuvant sunitinib therapy in the postsurgical setting of a kidney cancer model

**Fig. 1:**
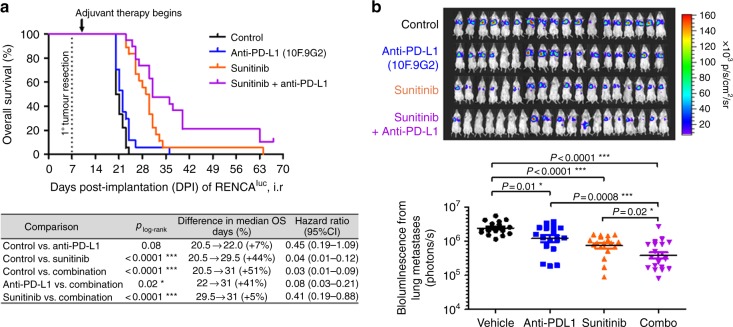
Combining an anti-PD-L1 antibody with sunitinib in the adjuvant (postoperative) therapy setting for renal cancer. At 7 days after intra-renal implantation of 10^5^ RENCA^luc^ cells, primary orthotopic (intra-renal (i.r.)) kidney tumours were surgically resected. Adjuvant therapies began on day 10 for sunitinib and its vehicle control, and on day 11 for the anti-PD-L1 antibody (clone 10F.9G2) and its isotype control. **a** Kaplan–Meier survival analyses and log-rank tests show PD-L1 inhibition prolonging overall survival when added to adjuvant sunitinib therapy. **b** Bioluminescent imaging of treatment effects on lung metastatic burden. The *P* values displayed are derived from unpaired *t* tests of log_10_-transformed thoracic bioluminescent fluxes measured from images taken between 19 and 21 days post implantation (this experiment was replicated three times, with *n* = 17–19 mice per treatment group in total; see Suppl. Fig. S1 for further details). Means ± SEM are shown

In several experiments where the RENCA^luc^ murine kidney cancer cell line was orthotopically implanted to create primary kidney tumours, adjuvant therapies began 3 to 4 days post resection (Fig. 1).

In the adjuvant setting, anti-PD-L1 monotherapy was ineffective, while sunitinib (VEGFR2 TKI) monotherapy was effective in terms of prolonging overall survival (OS) (Fig. 1a) and inhibiting the rapid postsurgical progression of RENCA^luc^ lung metastases (Fig. 1b). It should be noted that this preclinical model overestimates the clinical efficacy of single-agent sunitinib as adjuvant therapy for RCC—which thus far has not shown OS benefits but only an improvement in disease-free survival (DFS) in one of two randomised phase III clinical trials^30–33^—likely in part because the RENCA^luc^ model progresses so quickly that for some mice we are also modelling sunitinib therapy for advanced metastatic RCC rather than purely “authentic” adjuvant therapy for micro-metastatic disease (to be elaborated on in the Discussion). Nonetheless, the combination therapy—adjuvant anti-PD-L1 plus sunitinib—was also more effective than the individual drugs used as single agents, as measured by survival prolongation (Fig. 1a) or anti-metastatic activity (Fig. 1b). The degree of added benefit from combining sunitinib with anti-PD-L1 appeared to be greatest in the cohort of mice with the lowest rate of postsurgical primary tumour regrowth (Suppl. Fig. S1).

**Fig. 2:**
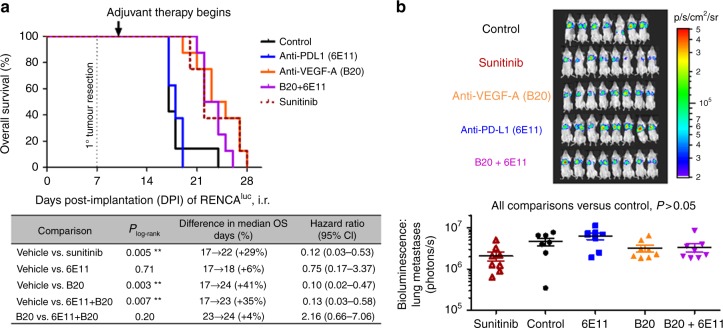
Combining an anti-PD-L1 antibody with an anti-VEGF-A antibody in the adjuvant (postoperative) therapy setting for renal cancer. At 7 days after intra-renal implantation of 10^5^ RENCA^luc^ cells, primary orthotopic (intra-renal (i.r.)) kidney tumours were surgically resected. Adjuvant therapies began on day 10 for the anti-VEGF-A antibody (clone B20-4.1.1) and on day 11 for the anti-PD-L1 antibody (clone 6E11), along with their respective isotype controls. **a** Kaplan–Meier survival analyses and log-rank tests show no difference in overall survival with the addition of PD-L1 inhibition to anti-VEGF-A therapy. **b** Bioluminescent imaging of treatment effects on lung metastatic burden. The *P* values displayed are derived from unpaired *t* tests of log_10_-transformed thoracic bioluminescent fluxes measured from images taken at day 16 post implantation (*n* = 7–8 mice per treatment group). Means ± SEM are shown

Similar to sunitinib, B20-4.1.1 (an anti-VEGF antibody; abbreviated as “B20” hereafter) was also effective as an adjuvant monotherapy (Fig. 2a, b). However, unlike sunitinib, adjuvant B20 therapy was not improved by the co-administration of an anti-PD-L1 antibody (Fig. 2a, b).

### Anti-PD-L1 plus sunitinib is ineffective in the unresected primary tumour and late-stage metastatic settings of a kidney cancer model

**Fig. 3:**
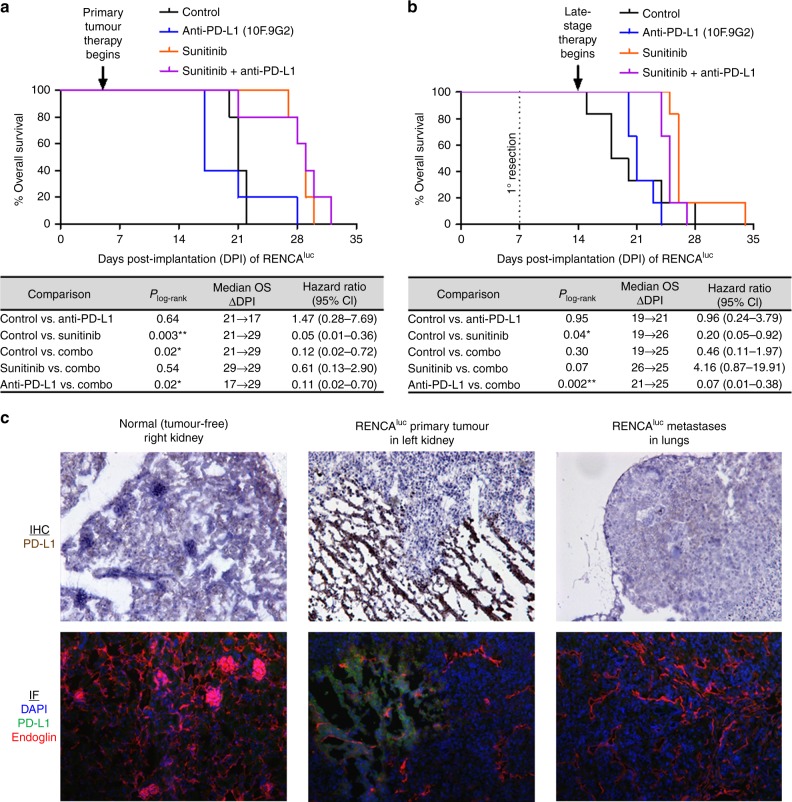
Sunitinib plus anti-PD-L1 combination therapy in the unresected primary tumour setting and postsurgical advanced metastatic disease setting of renal cancer. **a**, **b** 10^5^ RENCA^luc^ cells were orthotopically implanted on day 0. Kaplan–Meier survival analyses show that adding PD-L1 inhibition to sunitinib therapy does not improve survival either in the primary tumour therapy setting or in the late-stage therapy setting. **a** In the presence of unresected orthotopic primary kidney tumours, sunitinib and anti-PD-L1 (clone 10 F.9G2) therapies were started at 5 and 6 days post implantation (DPI) respectively. Three mice in the anti-PD-L1 monotherapy group experienced severe acute toxicity (laboured breathing and lethargy) immediately after administration of their 4th dose (17DPI), requiring immediate killing. **b** With the primary kidney tumours resected at 7 DPI, sunitinib (60 mg/kg/d) and anti-PD-L1 (clone 10F.9G2) treatments were initiated at 14 and 15 DPI respectively, i.e., after the establishment of advanced metastatic disease. **c** Tissues from the control group of the primary tumour therapy experiment (**a**) were collected at endpoint, sectioned, and subjected to IHC and IF staining for PD-L1; normal kidney tissue and lung metastases showed negligible positivity while the necrotic areas within RENCA^luc^ primary tumours were often positively stained; see Suppl. S3A for monochrome IF images

We next performed an experiment where primary RENCA^luc^ tumours were left unresected while systemic therapies were started early (at 5-6 DPI, as soon as primary tumours were beginning to establish) and were maintained as metastatic disease developed and progressed. In this setup, sunitinib monotherapy was still effective in prolonging survival and delaying lung metastases, but the addition of anti-PD-L1 yielded no additional benefit (Fig. 3a and Suppl. Fig. S2A). This was consistent with our observation, described in the previous section, that co-administered anti-PD-L1 was more effective at improving adjuvant sunitinib therapy when the rate of postsurgical primary tumour regrowth was lower. Notably, the presence of CD3^+^ T cells within primary RENCA^luc^ tumours was sparse, and among these, a considerable subset also expressed FoxP3, a marker of immunosuppressive Tregs (Suppl. Fig. S4).

We also performed an experiment where primary RENCA^luc^ tumours were resected but systemic therapies were delayed until advanced metastatic disease had established; i.e., beginning at 14 DPI, at which time the lung metastases had become macroscopic and clearly detectable by bioluminescent imaging (Suppl. Fig. S2B). Here, again, single-agent sunitinib was effective in prolonging survival (Fig. 3b). However, adding anti-PD-L1 to sunitinib yielded no additional survival benefit (Fig. 3b), despite seemingly improving metastatic control based on imaging (Suppl. Fig. S2B).

In considering why the addition of anti-PD-L1 improves sunitinib therapy only in the adjuvant setting (Fig. 1), but not in the primary tumour therapy setting (Fig. 3a), or in the late-stage metastatic disease setting (Fig. 3b), we asked whether PD-L1 expression might be higher in lung metastases versus their originating primary kidney tumours, as was reported to be the case in clinical patient samples.^43^ For this purpose, we collected the left kidneys which hosted RENCA^luc^ primary tumours, the normal right kidneys, and the lungs which contained extensive RENCA^luc^ metastases—from control mice—at the endpoint of the survival experiment involving unresected primary RENCA^luc^ tumours (Fig. 3a). Normal kidney tissue sections showed negligible histological staining for PD-L1 (Fig. 3c). Unexpectedly, endpoint RENCA^luc^ lung metastases also showed negligible PD-L1 staining, while patches of PD-L1 positivity were found within endpoint primary kidney tumours and usually coincided with necrotic areas (Fig. 3c). We should emphasise that endpoint lung metastases were analysed here, so it remains possible that PD-L1 expression might have been high within RENCA^luc^ lung metastases during earlier stages when adjuvant therapies were active.

### Anti-PD-L1 is by itself highly effective in the adjuvant setting of a breast cancer model

While the anti-PD-L1 plus sunitinib combination was effective as adjuvant therapy in the RENCA^luc^ model, we hypothesised we might get divergent results if we modelled a different cancer type—e.g., breast cancer. Clinically, for patients with metastatic breast cancer, sunitinib is not a therapeutic option, while bevacizumab remains an approved treatment in Europe.^36^ For this study, EMT-6/CDDP^40^—a cisplatin-resistant variant of the murine breast cancer cell line, EMT-6/P—was chosen because it yields higher incidences of lung metastases after orthotopic implantation and surgical resection compared to its parental line (100% vs. 50% of mice develop macroscopic lung metastases 4 weeks after primary tumour resection, data not shown), and it also shows higher basal PD-L1 expression than the parental line in vitro (Suppl. Fig. S5A).

**Fig. 4:**
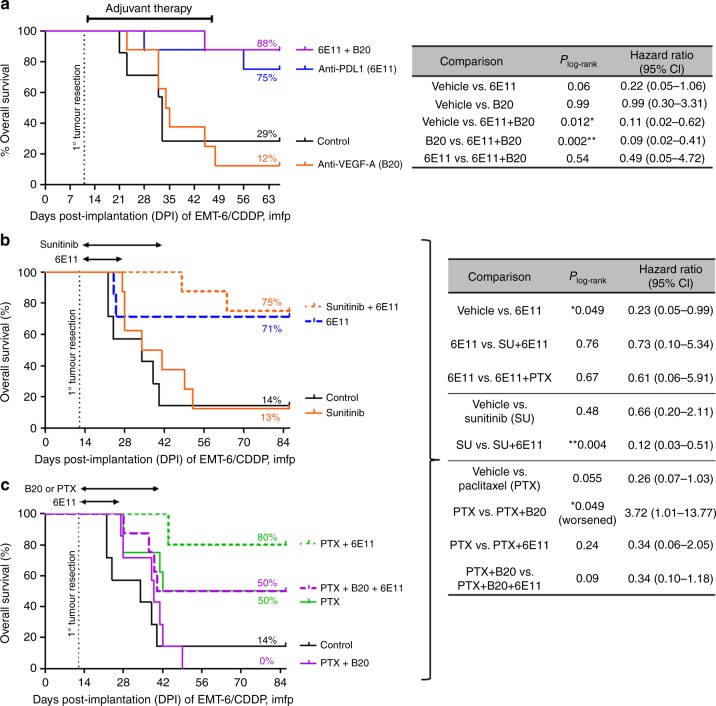
Combining anti-PD-L1 with antiangiogenic agents and/or chemotherapy in the adjuvant (postoperative) setting for breast cancer. Orthotopic (intra-mammary fat pad (imfp)) implantation of 2 × 10^5^ EMT-6/CDDP cells occurred on day 0. The resulting primary breast tumours were resected on day 11 (**a**) or day 12 (**b**, **c**, showing data from one experiment displayed across 2 panels). Adjuvant treatments began 1 day later, involving either 5 weeks (10 doses; **a**) or 2 weeks (4 doses; **b**) of the anti-PD-L1 antibody (clone 6E11); 4 weeks of sunitinib (**b**); 4 weeks of paclitaxel (**c**); 4 weeks of anti-VEGF antibody (clone B20-4.1.1; **a**, **c**). Kaplan–Meier survival curves are shown, along with *P* values derived from log-rank tests and hazard ratios for relevant comparisons

**Fig. 5:**
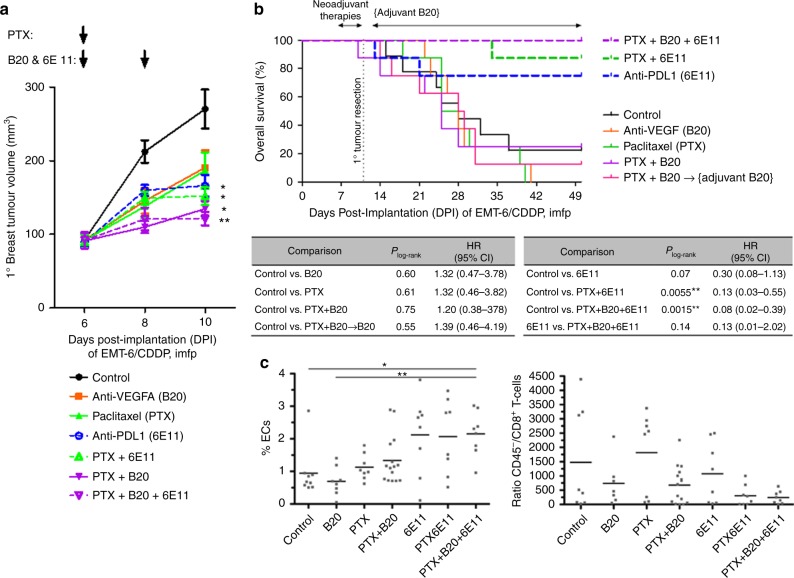
Combining anti-PD-L1 with chemotherapy, with or without anti-VEGF-A, in the neoadjuvant (preoperative) setting of breast cancer. At 6 days after the orthotopic implantation of 2 × 10^5^ EMT-6/CDDP cells, neoadjuvant therapies were administered according to the dosing schedules (black arrows) depicted above tumour growth curves in (**a**). Primary breast tumours were then resected on day 11 post implantation, after which only one group received adjuvant anti-VEGF-A (clone B20-4.1.1) therapy which resumed on day 13, as depicted over the Kaplan–Meier survival curves shown in (**b**). The resected primary breast tumours were dissociated and subjected to flow cytometry to quantify the % of VEGFR2^+^CD31^+^CD45^−^ endothelial cells (**c**) and the ratio of CD45^−^ non-immune cells (mostly tumour cells) vs. CD45^+^CD3^+^CD8^+^ T cells (**d**); *P < 0.05 and **P < 0.01 as calculated by the Kruskal–Wallis test and Dunn’s post-test; means are also depicted. See Suppl. Fig. S5C for other measures of postsurgical outcomes

Two adjuvant therapy experiments were performed using the orthotopic EMT-6/CDDP model (Fig. 4). Among the adjuvant monotherapies tested, single-agent B20 or sunitinib yielded no survival benefit (Fig. 4a, b respectively); paclitaxel (PTX) alone showed a trend of improving OS (Fig. 4c), while 6E11 alone markedly increased long-term survival rates (from 29% to 75% at 9 weeks post implantation in Fig. 4a; from 14% to 71% at 12 weeks in Fig. 4b). Among the combination therapies tested, the addition of B20 to adjuvant paclitaxel actually worsened OS (*P*_log-rank_ = 0.049; Fig. 4c), while the addition of 6E11 to either B20 or sunitinib improved OS (*P*_log-rank_ = 0.002 and 0.004 respectively; Fig. 4a, b). If 6E11—which was highly effective by itself—were to be the backbone of adjuvant combination therapy, the addition of B20, sunitinib, or PTX all slightly increased long-term survival rates (75% to 88% in Fig. 5a; 71% to 75% in Fig. 4b; 71% to 80% in Fig. 4c), but these slight improvements in OS were statistically non-significant (*P*_log-rank _> 0.05; Fig. 4).

### Co-administrating anti-PD-L1 improves neoadjuvant anti-VEGF plus paclitaxel chemotherapy in a breast cancer model

The above combinations were also tested as neoadjuvant therapies in the orthotopic EMT-6/CDDP model. In contrast to the orthotopic RENCA model, PD-L1 is widely and highly expressed in vivo within EMT-6/CDDP primary breast tumours and their associated lung metastases (Suppl. Fig. S3B).

Preoperative treatment with PTX+B20 (paclitaxel chemotherapy plus anti-VEGF-A) effectively suppressed primary breast tumour growth compared to controls (Fig. 5a), but this did not translate into any appreciable improvement in OS (Fig. 5b). In contrast, two neoadjuvant therapy combinations containing 6E11 (anti-PD-L1)—specifically, PTX+6E11 and PTX+B20+6E11—resulted in effective presurgical suppression of primary breast tumour growth (*P* < 0.05, Fig. 5a) as well as significant postsurgical OS benefits (*P*_log-rank _< 0.05; Fig. 5b).

Immediately after surgical resection of these neoadjuvantly treated primary EMT-6/CDDP breast tumours, they were dissociated into single-cell suspensions and subjected to flow cytometry analysis. Out of the total viable cells, few were CD45^−^VEGFR2^+^CD31^+^ ECs (<4%; Fig. 5c), while about 20–30% on average were CD45^+^VEGFR2^−^CD31^−^ immune cells (Fig. 5d). Neoadjuvant B20 treatment led to a slight decrease in intratumoural EC content that was not statistically significant (*P* > 0.05, Fig. 5c). The triple combination of PTX+B20+6E11, however, led to a statistically significant increase in intratumoural EC content compared to controls (from 1% to 2%, *P* < 0.05, Fig. 5c)—possibly reflective of decreased tumour cell content from effective tumour cell kill. While there were no statistically significant differences between treatment groups in the intratumoural ratio of CD45^−^ cells versus CD8^+^ T cells (which is roughly a measure of tumour burden divided by tumour-infiltrating cytotoxic T cells), the greatest reduction in the mean of this ratio was achieved by the triple combination therapy of PTX+B20+6E11 compared to controls (*P* < 0.05, Fig. 5d).

## Discussion

For over a decade, we have developed a number of preclinical models for evaluating experimental therapeutics in mice as postsurgical treatments of either early-stage microscopic metastatic disease or more advanced, overt, metastatic disease.^41,44–47^ The rationale was to improve the predictive potential of preclinical testing of new drugs/therapeutics before they are evaluated in clinical trials involving patients with either early- or late-stage metastatic disease. Studies conducted using such preclinical models, for example, retrospectively recapitulated the negative phase 3 clinical trial outcomes for antiangiogenic drugs such as sunitinib in metastatic breast cancer^22^ and also mirrored the inadequacies of VEGF/VEGFR2 pathway inhibitors in general as adjuvant treatments across multiple indications.^19,34,35^ Our earlier preclinical models all involved the growth and metastatic spread of human tumour xenografts in immunosuppressed mice, which did not therefore allow similar studies to be undertaken for immunotherapies, including immune checkpoint inhibitors such as PD-L1 antibodies. Thus, we developed several new models involving the orthotopic growth of syngeneic mouse tumour cell lines in immunocompetent mice and surgical resection of primary tumours before systemic postsurgical therapies, as reported here and elsewhere.^34^

In this study, we evaluated the in vivo efficacy of several clinically relevant combinations of VEGF/VEGFR2 inhibitors and PD-L1 inhibitors when administered at different stages of disease in two syngeneic murine tumour models. In the orthotopic RENCA^luc^ kidney cancer model—where in vivo PD-L1 expression was almost negligible within the primary tumour and sparse within lung metastases—sunitinib plus anti-PD-L1 was an effective combination in the adjuvant therapy setting but not in the primary tumour or advanced metastatic disease settings. In the orthotopic EMT-6/CDDP breast cancer model—where in vivo PD-L1 expression was widespread within primary tumours and even more prevalent within lung metastases—single-agent anti-PD-L1 was highly effective in the adjuvant therapy setting, while combining anti-PD-L1 with paclitaxel chemotherapy was beneficial in the neoadjuvant therapy setting. Our results emphasise the value and need for preclinically evaluating therapies in early- or late-stage metastatic disease, in addition to or even instead of in primary tumours. We observed multiple examples of divergent therapy outcomes depending on the stage of disease that existed when therapy was initiated, as summarised below.

I. Sunitinib plus anti-PD-L1 is a promising combination adjuvant therapy for kidney cancer
  : Although cultured RENCA cells expressed high basal levels of PD-L1 in vitro, their in vivo PD-L1 expression was heterogeneous within endpoint RENCA^luc^ orthotopic primary kidney tumours (usually coincided with areas of necrosis) and almost absent in endpoint RENCA^luc^ lung metastases. The sparsity of in vivo PD-L1 expression likely explains the ineffectiveness of single-agent anti-PD-L1 (10F.9G2 or 6E11) in vivo across all treatment settings tested in the RENCA^luc^ model. In the case of 10F.9G2, acute drug toxicity—in the form of sudden-onset dyspnoea and lethargy, requiring immediate culling— could also have limited its potential efficacy as monotherapy. These acute adverse reactions were sometimes observed immediately after administration of the fourth dose of 10F.9G2 (see Suppl. Figs. S1 and S2), while 6E11 could be given for the entire duration of survival experiments without causing similar acute toxicities (up to 5 doses in the adjuvant RENCA model as in Fig. 2 and up to 10 doses in the adjuvant EMT-6/CDDP model as in Fig. 4a).
  In contrast, single-agent sunitinib effectively extended OS in all three settings—as adjuvant therapy immediately post resection of primary renal tumours, as late-stage therapy for advanced metastatic disease with resected primary renal tumours, or as early therapy for unresected primary tumours. The RENCA^luc^ model accurately recapitulates the clinical efficacy of sunitinib as a treatment for advanced mRCC, but overestimates the clinical efficacy of sunitinib as an adjuvant therapy, which thus far has only shown an improvement in DFS in one of two randomised phase 3 trials involving resectable RCC (only in the “S-TRAC” trial^32,33^ but not in the “ASSURE” trial^30,31^), without yielding OS benefits in either trial. Our overestimation is likely due to the aggressiveness of the RENCA^luc^ model—with lung metastases quickly progressing from microscopic stage at ~10 DPI to macroscopic stage at ~14 DPI—and the increased responsiveness of more advanced disease to sunitinib treatment. Interestingly, the co-administration of anti-PD-L1 with sunitinib yielded additional benefit only in the adjuvant therapy setting. This raises the possibility that PD-L1 expression could have been transiently higher in early-stage RENCA^luc^ lung metastases compared to endpoint, although this was not directly assessed. Unlike with sunitinib, adjuvant anti-VEGF (B20) therapy was not improved by combination with an anti-PD-L1 antibody. A caveat limiting this comparison was that different murine PD-L1-specific antibodies were used—sunitinib was combined with clone 10F.9G2 (a rat IgG2b) while B20 was combined with clone 6E11 (a mouse IgG1)—but at similar doses (fixed 100 μg/dose vs. 100 μg/20 g respectively).
  To our knowledge, there is currently only one phase 3 trial underway to evaluate anti-PD-L1 therapy in the adjuvant setting of resectable RCC: the “IMmotion010” trial comparing single-agent atezolizumab vs. placebo (ClinicalTrials.gov identifier: NCT03024996). Our RENCA^luc^ model predicts limited efficacy of such adjuvant anti-PD-L1 monotherapy, and instead suggests more promising combination activity when combined with sunitinib. This potential combination is worthy of further study particularly in light of the recent FDA approval of sunitinib as adjuvant therapy for patients with high-risk RCC post nephrectomy.^37^
II. Neoadjuvant anti-PD-L1 plus paclitaxel chemotherapy and adjuvant anti-PD-L1 therapy for resectable breast cancer:
  Although cultured EMT-6/CDDP cells expressed only moderate basal levels of PD-L1 in vitro, their in vivo PD-L1 expression was widespread within resected EMT-6/CDDP orthotopic primary breast tumours and abundant in endpoint EMT-6/CDDP lung metastases. Unsurprisingly then, anti-PD-L1 (6E11) by itself was highly effective as an adjuvant therapy in the EMT-6/CDDP model in terms of prolonging OS, while the addition of B20, sunitinib, or PTX to adjuvant anti-PD-L1 therapy only modestly improved long-term survival rates. To our knowledge, there is currently only one phase 3 trial underway to evaluate anti-PD-L1 (avelumab) therapy in the adjuvant setting of resectable TNBC (Suppl. Table 3: the “A-Brave” trial). Given the oestrogen receptor-negative status of the cisplatin-resistant EMT-6/CDDP cell line,^48^ our results would predict significant OS benefit with adjuvant avelumab therapy at least in the subset of patients with PD-L1-high TNBCs. Conceivably, for PD-L1-low breast cancers, combination activity may be greater than what is predicted by the EMT-6/CDDP model, with PTX being a more promising partner than sunitinib or B20 for combining with adjuvant anti-PD-L1 therapy. Also of note in the adjuvant EMT-6/CDDP model, B20 alone had no effect on OS while the addition of B20 to paclitaxel chemotherapy actually reduced OS. In comparison, the addition of bevacizumab to adjuvant chemotherapies has not led to any DFS or OS benefits, but also did not worsen OS, in three adjuvant phase 3 trials involving resectable breast cancer.^49–51^
  In the neoadjuvant therapy setting of the EMT-6/CDDP model, PTX+B20 effectively suppressed primary breast tumour growth before surgery but did not yield a lasting OS benefit. This mirrored the “GeparQuinto/GBG-44” phase 3 clinical trial, in which the addition of bevacizumab to neoadjuvant chemotherapy yielded an improvement in pathological complete response rates (pCR) in the TNBC subgroup,^52^ but with no accompanying 3-year DFS or OS benefit.^53^ The only neoadjuvant therapies that significantly prolonged OS in the EMT-6/CDDP model were PTX+6E11(±B20). Moreover, the triple combination of PTX+6E11+B20 increased intratumoural EC content compared to controls and resulted in the lowest tumour burden to infiltrating CD8+ T-cell ratio among the treatment groups tested. There are at least two phase 3 trials underway to evaluate the addition of atezolizumab to neoadjuvant chemotherapy for TNBC (Suppl. Table 3: “NeoTRIPaPDL1” and “IMpassion031”). Our EMT-6/CDDP data, if clinically translatable, predict that both of these trials will be successful.
III. Limitations and summary: We acknowledge several weaknesses or deficiencies in this study, several of which stem from the use of single cancer cell lines to model breast cancer and RCC, in part due to the limited number of murine cancer cell lines available for preclinical testing (in contrast to the diversity of human cancer cell lines). As discussed above, the aggressiveness of the RENCA^luc^ model makes it difficult to delineate micro- vs. macro-metastatic disease in the postoperative therapy setting and thus overpredicts the efficacy of adjuvant sunitinib. RENCA^luc^ is further limited by its wild-type VHL status, given that the majority of RCCs in human patients are of the clear-cell histological classification, which is highly associated with VHL mutations. Preclinical immuno-oncology drug testing has almost universally relied on RENCA as the single syngeneic model of RCC. A recent study described an attempt at CRISPR/Cas9-mediated knockout of VHL in RENCA, but the resulting variant suffered from variable tumour take and immune-mediated rejection after implantation in BALB/c mice.^54^ With regards to the EMT-6/CDDP model, this particular variant was chosen for its increased incidence of postsurgical metastases compared to its parental line, as mentioned above. Additionally, while the EMT-6 parental line is oestrogen receptor (ER)-positive, the EMT-6/CDDP variant is ER-negative,^48^ making it a better representation of ‘triple-negative breast cancers’—the subtype that has historically responded the most promisingly to antiangiogenic VEGF inhibitors in clinical trials of advanced metastatic breast cancers.^35^ Another limitation of the current preclinical study is its limited ability to model the full spectrum of toxicities that have been clinically associated with anti-PD-L1 agents^55^ and the potential added toxicities when such drugs are combined with antiangiogenic drugs. Toxicity from combination therapy is important to consider especially in the adjuvant therapy setting where the toxicities and side effects of an antiangiogenic drug alone have in some cases been deemed to outweigh its clinical benefits.^38^

In summary, preclinical models of metastatic cancers—especially those that recapitulate clinically relevant therapeutic settings (i.e., distinctly modelling neoadjuvant or adjuvant therapies for early-stage resectable cancers vs. late-stage therapies for advanced metastatic disease)—can be helpful in identifying the most promising combination therapies for translation into clinical trial testing. Using an RCC model that involved surgical resection of primary kidney tumours derived from orthotopic implantation of the RENCA^luc^ cell line, this study showed no prolongation of OS with adjuvant anti-PD-L1 monotherapy compared to controls, while the adjuvant combination of sunitinib and anti-PD-L1 improved OS compared to either drug alone. In the same RCC model, the addition of anti-PD-L1 to a bevacizumab-like agent in the adjuvant setting did not yield similar combination activity, while the sunitinib plus anti-PD-L1 combination became ineffective when administered as early therapy for unresected primary tumours or late-stage therapy for advanced metastatic disease. In a breast cancer model that involved surgical resection of primary breast tumours derived from orthotopic implantation of the EMT-6/CDDP cell line, anti-PD-L1 alone was highly effective as an adjuvant therapy, while the combination of anti-PD-L1 plus paclitaxel chemotherapy (with or without anti-VEGF) was found to be the most effective in the neoadjuvant therapy setting. Therefore, beyond the combinations that are currently being investigated in ongoing clinical trials (Suppl. Tables 2 and 3), our preclinical study additionally suggests that sunitinib plus anti-PD-L1 may be worthy of further investigation in the adjuvant setting for resectable RCC, while the potential for increased drug toxicities should also be considered.

## Electronic supplementary material

Supplemental Figures and Tables

## Data Availability

The raw datasets analysed for this study are available from the corresponding author upon request.
