# Supplementary material for: Pre- and post-operative anti-PD-L1 plus anti-angiogenic therapies in mouse breast or renal cancer models of micro- or macro-metastatic disease
Source: Br J Cancer. 2018 Nov 30;120(2):196–206. doi: 10.1038/s41416-018-0297-1 (PMC6342972; doi:10.1038/s41416-018-0297-1)
Supplement: Supplementary file 1 — Supplemental Figures and Tables [file 41416_2018_297_MOESM1_ESM.pdf]

Supplemental Information for:

Pre- and post-operative anti-PD-L1 plus anti-angiogenic therapies in mouse breast or renal cancer models of micro- or macro-metastatic disease

Florence T.H. Wu[1,2], Ping Xu[2], Annabelle Chow[2], Shan Man[2], Janna Krüger[2], Kabir A. Khan[2], Marta Paez-Ribes[2,3], Elizabeth Pham[2,4], and Robert S. Kerbel[1,2]*

[1]Department of Medical Biophysics, University of Toronto, Toronto, Ontario, Canada

[2]Biological Sciences Platform, Sunnybrook Research Institute, Toronto, Ontario, Canada

[3]Current: Department of Oncology, University of Cambridge, Hutchison/MRC Research Centre, Cambridge, UK

[4]Current: Amgen Discovery Research, South San Francisco, California, USA

*Corresponding author: robert.kerbel@sri.utoronto.ca

Table of Contents

Supplemental Figure S1. Subgroup analyses from the RENCA^{luc} experiment evaluating adjuvant anti-PD-L1 plus sunitinib combination therapy. 2

Supplemental Figure S2. Serial bioluminescent imaging from RENCA^{luc} experiments evaluating anti-PD-L1 plus sunitinib therapy in non-adjuvant settings. 3

Supplemental Figure S3. Specificity of IHC and IF staining of mouse PD-L1. 4

Supplemental Figure S4. Immunofluorescent staining of T cells in RENCA^{luc} primary kidney tumors versus spleen controls. 6

Supplemental Figure S5. Additional data from the EMT-6/CDDP experiments. 7

Supplemental Table 1. U.S. Food and Drug Administration (FDA)-approved indications for immune checkpoint inhibitors directed against PD-1 or PD-L1, as of July 2018. 8

Supplemental Table 2. Ongoing Phase 3 clinical trials testing combinations of anti-PD-L1 plus antiangiogenic agents in inoperable late-stage disease settings. 9

Supplemental Table 3. Ongoing Phase 3 clinical trials testing anti-PD-L1 agents in the adjuvant or neoadjuvant therapy settings. 10

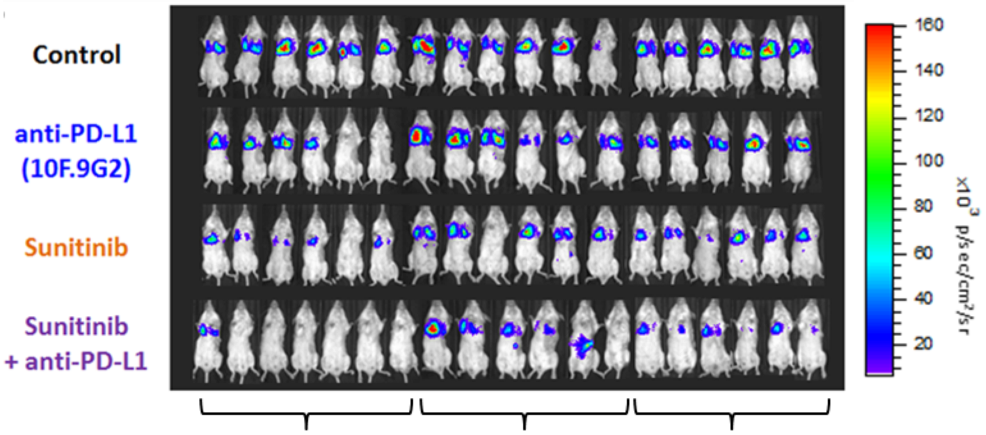

	Subgroup "15-316"	Subgroup "15-321"	Subgroup "15-316B"	Overall Analysis
**Sex of BALB/c mice:**	Male	Female	Male	**M:F = 68%:32%**
**Day of Imaging:** (DPI = days post-implantation)	19 DPI	19 DPI	21 DPI	**19-21 DPI**
**Rate of post-surgical regrowth at site of 1° kidney tumors:**	8% (2/25)	48% (11/23)	12% (3/25)	**22% (16/73)**
**Interruptions in sunitinib treatment due to toxicity** (e.g., 10-15% weight loss)	Switched to 2dOFF/5dON cycles at 51 DPI (applied to 5 surviving mice)	None (maintained until the end, 40 DPI)	None (maintained until the end, 36 DPI)	**7% (5/73)**
**Interruptions in αPDL1 treatment due to toxicity** (e.g., acute dyspnea & lethargy)	Break after 4 doses from 25 DPI to 55DPI (applied to 12 surviving mice)	None (maintained until the end, 40 DPI)	None (maintained until the end, 36 DPI)	**16% (12/73)**
**Kaplan-Meier Analysis of Overall Survival Differences:** Hazard Ratios (95% confidence intervals)				
**Control vs. αPDL1+Sunitinib**	0.02 (0.003-0.20)	0.04 (0.01-0.28)	0.04 (0.01-0.25)	**0.03 (0.01-0.09)**
**Sunitinib vs. αPDL1+Sunitinib**	0.28 (0.07-1.13)	0.68 (0.18-2.50)	0.50 (0.11-2.22)	**0.41 (0.19-0.88)**
**αPDL1 vs. αPDL1+Sunitinib**	0.06 (0.01-0.34)	0.03 (0.004-0.25)	0.05 (0.01-0.30)	**0.08 (0.03-0.21)**

Supplemental Figure S1. Subgroup analyses from the RENCA^{luc} experiment evaluating adjuvant anti-PD-L1 plus sunitinib combination therapy.

Inter-experimental variability within the aggregated data shown in Figure 1.

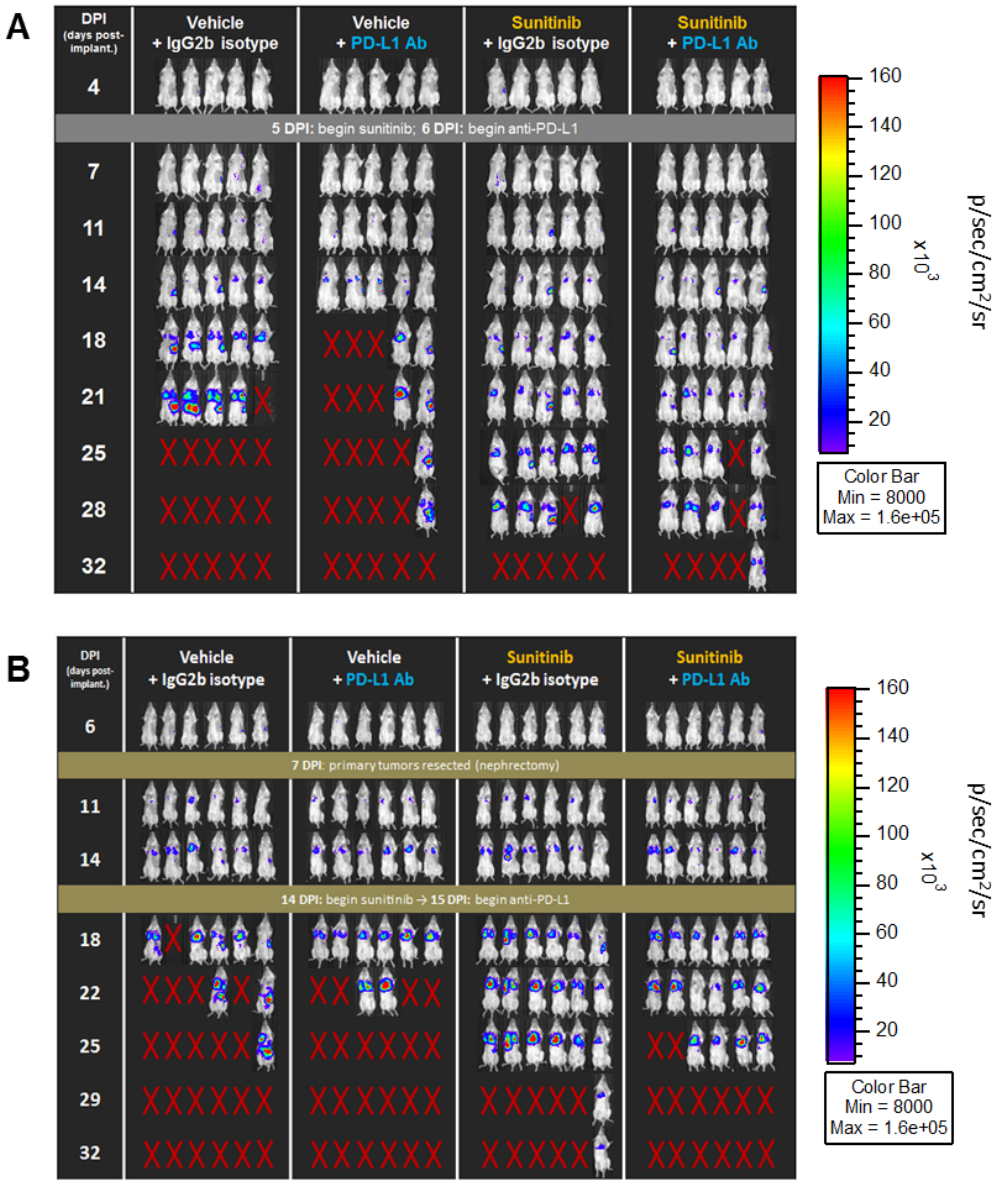

<u>Supplemental Figure S2</u>. Serial bioluminescent imaging from RENCA^{luc} experiments evaluating anti-PD-L1 plus sunitinib therapy in non-adjuvant settings.

A: Bioluminescent data from the experiment shown in Figure 3A which did not involve resection of primary kidney tumors. Sunitinib and anti-PD-L1 therapies were started at 5 and 6 days post-implantation (DPI) respectively. Three mice in the anti-PD-L1 monotherapy group experienced severe acute toxicity (labored breathing and lethargy) immediately after administration of their 4th dose (17DPI), requiring immediate euthanasia. **B**: Bioluminescent data from experiment shown in Figure 3B, where primary kidney tumors were resected. Sunitinib and anti-PD-L1

therapies were initiated at 14 and 15 DPI respectively, i.e., in the advanced stages of postsurgical metastatic disease.

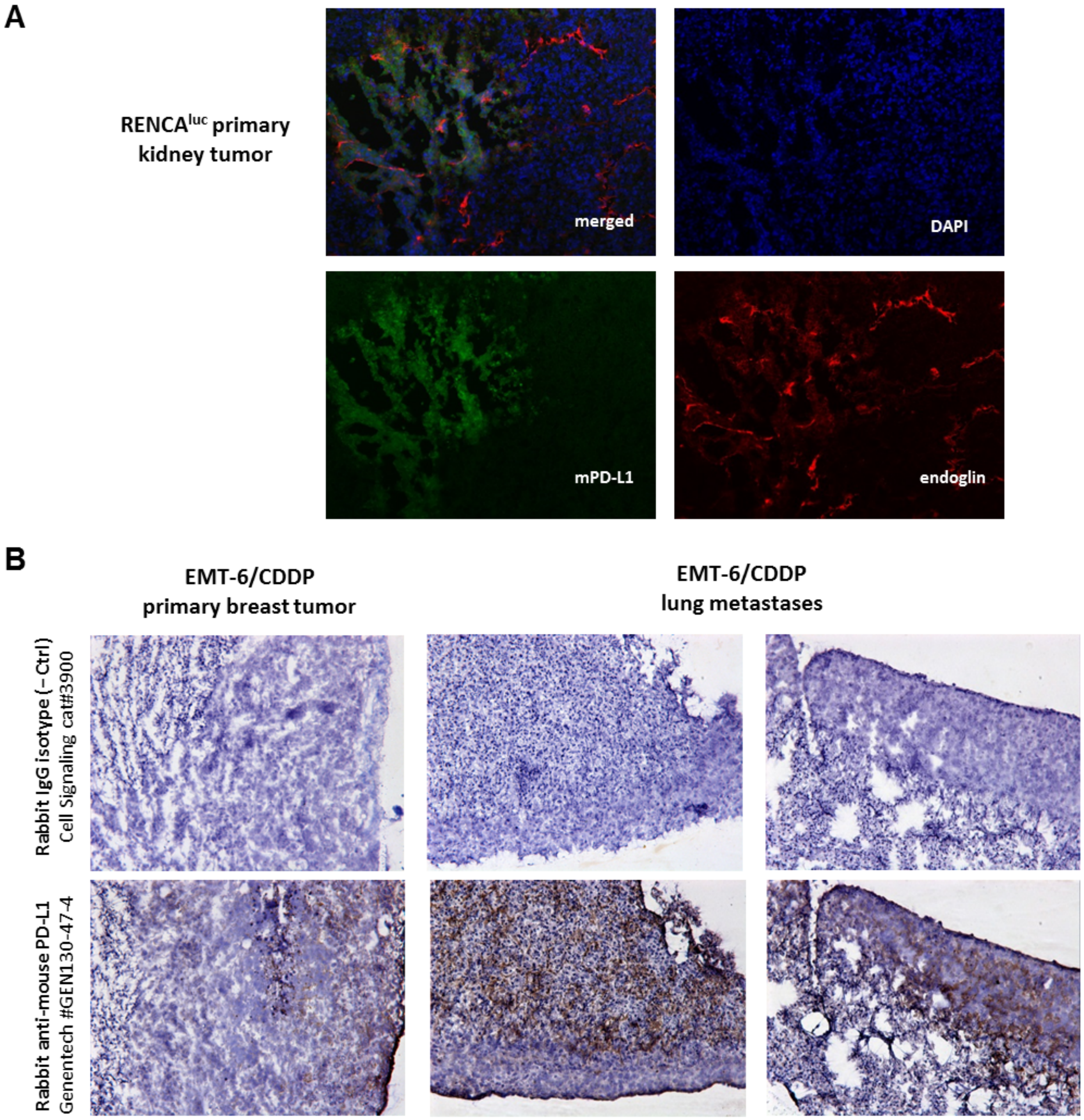

Supplemental Figure S3. Specificity of IHC and IF staining of mouse PD-L1.

A: Juxtaposing the merged 3-color IF image from Figure 3C – co-staining a section of RENCA^{luc} primary kidney tumor for mouse PD-L1, mouse endoglin, and DAPI – with the individual monochrome images. **B**: Consecutive sections of EMT-6/CDDP primary breast tumors and lung

metastases subjected to IHC staining using a rabbit anti-mouse PD-L1 antibody (GEN130-47-4) versus a rabbit Ig isotype control (Cell Signaling cat#3900) as the primary antibody.

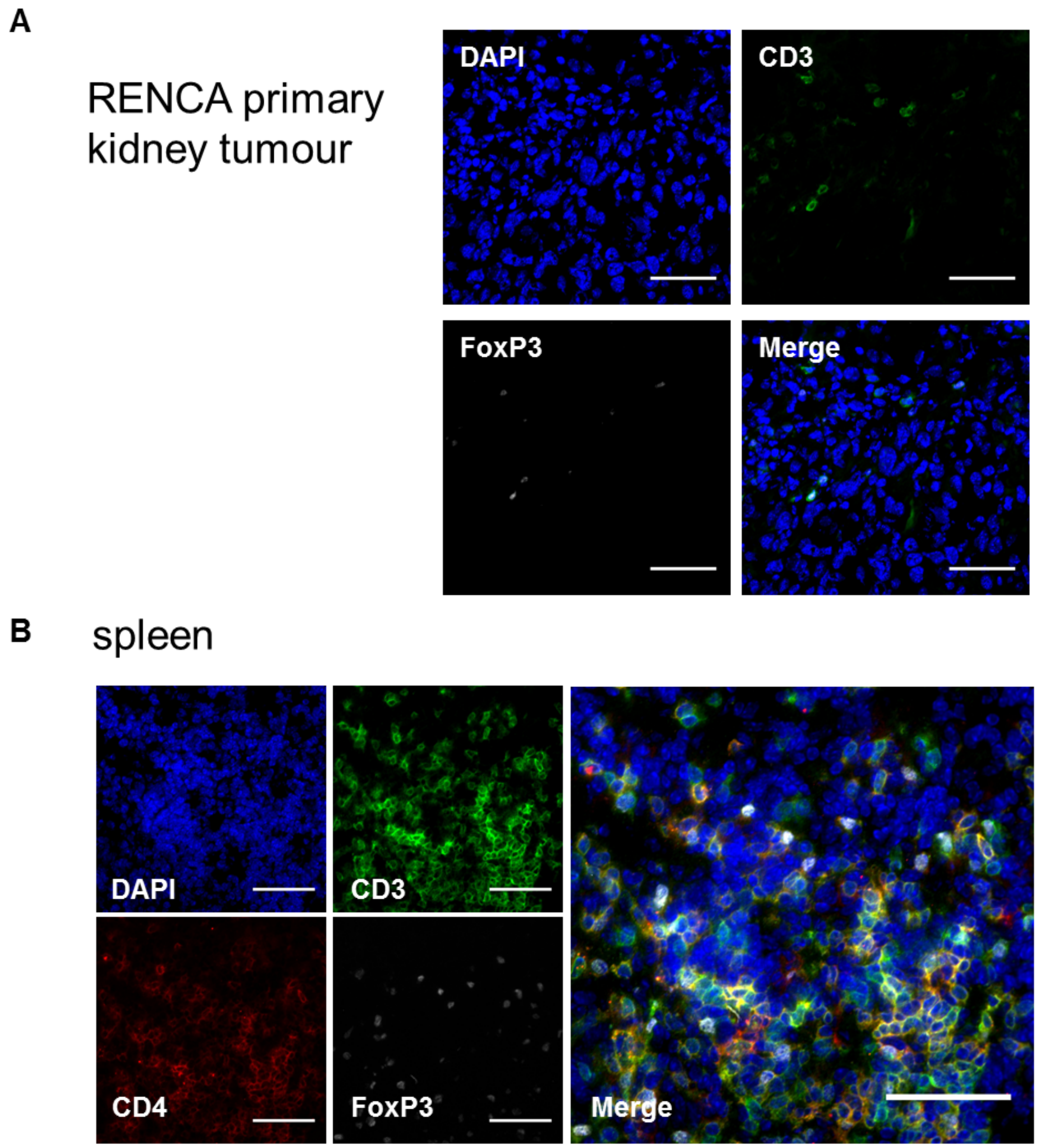

<u>Supplemental Figure S4</u>. Immunofluorescent staining of T cells in RENCA^{luc} primary kidney tumors versus spleen controls.

Acetone:methanol-fixed frozen sections of RENCA^{luc} primary kidney tumors (**A**) and spleens as control tissues (**B**) from tumor-bearing BALB/cJ mice were stained with the following primary antibodies: rabbit anti-CD3 (abcam clone SP7, cat# ab16669, 1:50 dilution), rat APC conjugated anti-FoxP3 (eBiosciences clone FJK-16s, cat# 17-5773-80, 1:40 dilution), with or without a rat biotinylated anti-CD4. Representative images are shown here, cropped and brightness enhanced on ImageJ. Scale bar 50μm.

A

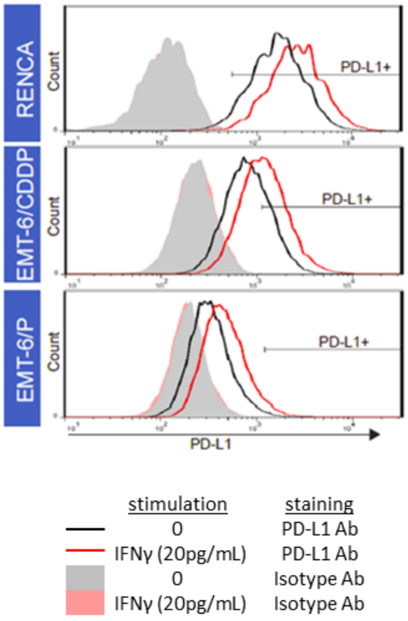

B

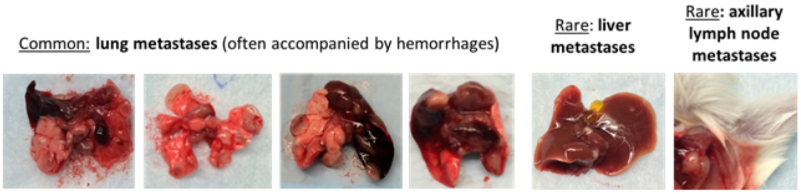

C

<table>
<tr><th>Group:</th><th>1</th><th>2</th><th>3</th><th>4</th><th>5</th><th>6</th><th>7</th><th>8</th></tr>
<tr><td>Neoadjuvant therapy:</td><td>-</td><td>B20</td><td>PTX</td><td>PTX +B20</td><td>PTX +B20</td><td>6E11</td><td>PTX +6E11</td><td>PTX +B20 +6E11</td></tr>
<tr><td rowspan="2">1° tumors invading into abdominal wall ‡
at the time of resection</td><td rowspan="2">6/9 (1.4)</td><td rowspan="2">3/8 (0.8)</td><td rowspan="2">2/8 (0.4)</td><td>2/8 (0.5)</td><td>4/8 (1.1)</td><td rowspan="2">5/8 (1.3)</td><td rowspan="2">2/8 (0.6)</td><td rowspan="2">4/8 (1.0)</td></tr>
<tr><td colspan="2">6/16 (0.8)</td></tr>
<tr><td>Adjuvant therapy:</td><td>-</td><td>-</td><td>-</td><td>-</td><td>B20</td><td>-</td><td>-</td><td>-</td></tr>
<tr><td>Regrowths at 1° tumor site</td><td>2/8</td><td>4/8</td><td>3/8</td><td>2/7♦</td><td>2/8</td><td>1/8</td><td>0/8</td><td>0/8</td></tr>
<tr><td>Ascites</td><td>2/8</td><td>0/8</td><td>1/8</td><td>0/8</td><td>0/8</td><td>0/8</td><td>0/8</td><td>0/8</td></tr>
<tr><td>Right axillary lymphatic metastasis</td><td>2/8</td><td>3/8</td><td>1/8</td><td>2/8</td><td>3/8</td><td>2/8</td><td>1/8</td><td>0/8</td></tr>
<tr><td>Lung metastases</td><td>6/8</td><td>8/8</td><td>6/8</td><td>5/8</td><td>7/8</td><td>1/8</td><td>1/8</td><td>0/8</td></tr>
<tr><td>Lung hemorrhaging</td><td>4/8</td><td>4/8</td><td>5/8</td><td>5/8</td><td>3/8</td><td>1/8</td><td>0/8</td><td>0/8</td></tr>
<tr><td>Long-term survival (alive at 50 DPI)</td><td>2/8</td><td>0/8</td><td>0/8</td><td>2/8</td><td>1/8</td><td>6/8</td><td>7/8</td><td>8/8</td></tr>
</table>

‡ Fractions denote incidence per group, e.g., "6/9" represents "6 mice out of 9". Each mouse was assigned an invasiveness score between 0 and 5, with 0 representing no signs of invasion and 5 representing a substantially invaded tumor. The average invasiveness score per group is shown within brackets. ♦ 1 mouse reached endpoint before resection day.

<u>Supplemental Figure S5</u>. Additional data from the EMT-6/CDDP experiments.

A: Flow cytometry detection of PD-L1 expression on murine kidney cancer cells (RENCA) and murine breast cancer cells (parental EMT-6/P and cisplatin-resistant EMT-6/CDDP) with or without IFNγ stimulation (20pg/mL). **B**. The orthotopically implanted EMT-6/CDDP model, especially when post-surgical resection of the established primary breast tumors, generates lung metastases and occasionally also liver and lymphatic metastases. **C**: Additional data from the neoadjuvant therapy experiment (Figure 5). The 6E11-containing treatments had the greatest long-term impact in terms of reducing postsurgical metastatic spread and improving long-term survival rates.

<u>Supplemental Table 1</u>. U.S. Food and Drug Administration (FDA)-approved indications for immune checkpoint inhibitors directed against PD-1 or PD-L1, as of July 2018.

Indications	anti-PD-1 antibodies		anti-PD-L1 antibodies		
	Pembrolizumab (Keytruda)	Nivolumab (Opdivo)	Atezolizumab (Tecentriq)	Avelumab (Bavencio)	Durvalumab (Imfinzi)
melanoma	✓	✓*			
non-small-cell lung cancer (NSCLC)	✓	✓	✓		✓
renal cell carcinoma (RCC)		✓			
urothelial carcinoma (UC)	✓	✓	✓	✓	✓
squamous cell carcinoma of the head and neck (SCCHN)	✓	✓			
classical Hodgkin lymphoma (cHL)	✓	✓			
hepatocellular carcinoma (HCC)		✓			
microsatellite instability-high (MSI-H) or mismatch repair-deficient (dMMR) solid tumors	✓				
MSI-H or dMMR colorectal cancer	✓	✓			
PD-L1-expressing gastric or gastroesophageal junction (GEJ) adenocarcinomas	✓				
Merkel cell carcinoma (MCC)				✓	
PD-L1-expressing cervical cancer	✓				
Primary mediastinal large B-Cell Lymphoma (PMBCL)	✓				

<u>Note</u>: All indications here refer to advanced (metastatic/inoperable) disease settings, with the exception indicated by an asterisk (*) where nivolumab is additionally approved as adjuvant treatment after surgical resection of melanoma.

Supplemental Table 2. Ongoing Phase 3 clinical trials testing combinations of anti-PD-L1 plus antiangiogenic agents in inoperable late-stage disease settings.

Phase 3 Trial Identifier	Interventions	Conditions
NCT02684006 (JAVELIN Renal 101)	**avelumab**+axitinib vs. sunitinib	Advanced or metastatic renal cell carcinoma (mRCC)
NCT02420821 (IMmotion151)	**atezolizumab**+bevacizumab vs. sunitinib	
NCT02366143 (IMpower150)	{**atezolizumab**+bevacizumab vs. **atezolizumab** vs. bevacizumab} + paclitaxel+carboplatin	Stage IV non-squamous non-small cell lung cancer (NSCLC)
NCT02891824 (ATALANTE)	{**atezolizumab** vs. placebo} + bevacizumab + platinum-based chemotherapy	Late-relapse or recurrent ovarian, fallopian tube, or primary peritoneal cancer (Ov/FT/PPC)
NCT02839707 (NRG-GY009)	{**atezolizumab**+bevacizumab vs. **atezolizumab** vs. bevacizumab} + pegylated liposomal doxorubicin	

Note: Anti-PD-L1 agents are **bolded**. Chemotherapies are in green. Antiangiogenics are in orange.

<u>Supplemental Table 3</u>. Ongoing Phase 3 clinical trials testing anti-PD-L1 agents in the adjuvant or neoadjuvant therapy settings.

Phase 3 Trial	Interventions	Conditions
Neoadjuvant therapy in Preoperative Setting		
NCT02620280 (NeoTRIPaPDL1)	Carboplatin + Nab-paclitaxel + **Atezolizumab** vs. Carboplatin + Nab-paclitaxel	Invasive ductal TNBC
NCT03197935 (IMpassion031)	Nab-paclitaxel + {**Atezolizumab** vs. placebo}	Early-stage TNBC
NCT03038100 (IMagyn050)	{**Atezolizumab** vs. placebo} + paclitaxel + carboplatin + bevacizumab	Stage 3 or 4 Ov/FT/PPC
Neoadjuvant and Adjuvant therapy in Perioperative Setting		
NCT03281954 (NSABP B-59)	{**atezolizumab** vs. placebo} + paclitaxel + carboplatin → {**atezolizumab** vs. placebo} + doxorubicin/epirubicin + cyclophosphamide → Surgery → {**atezolizumab** vs. placebo}	TNBC
Adjuvant therapy in Postoperative Setting		
NCT02450331 (IMvigor010)	**Atezolizumab** vs. Observation	Muscle-invasive urothelial carcinoma
NCT03024996 (IMmotion010)	**Atezolizumab** vs. Placebo	Renal cell carcinoma (RCC)
NCT02912559	FOLFOX + **Atezolizumab** vs. FOLFOX	Stage III colon cancer with deficient DNA mismatch repair (dMMR)
NCT03271372	**Avelumab** vs. Placebo	Merkel Cell Carcinoma
NCT02926196 (A-Brave)	**Avelumab** vs. Observation	Triple-negative breast cancer (TNBC)
NCT02273375	**Durvalumab** vs. Placebo	Non-small-cell lung cancer (NSCLC)
NCT02486718	cisplatin-based chemotherapy → {**atezolizumab** vs. best supportive care}	Stage IB-IIIA NSCLC
NCT03038100 (IMagyn050)	{**Atezolizumab** vs. placebo} + paclitaxel + carboplatin + bevacizumab	Stage 3 or 4 Ovarian, Fallopian Tube, or Primary Peritoneal Cancer (Ov/FT/PPC)

<u>Note</u>: Anti-PD-L1 agents are **bolded**. Chemotherapies are in green. Antiangiogenics are in orange.
